# Acepromazine Reduces Airway Contraction in a Murine Model of Asthma

**DOI:** 10.1111/1440-1681.70112

**Published:** 2026-02-23

**Authors:** Alembert Lino‐Alvarado, Maria Aparecida de Oliveira, Carmen Silvia Moreno Serra, Isabella Victoria Santana Melhado, Rafael Campos, Yanira Riffo‐Vasquez, Henrique Takachi Moriya

**Affiliations:** ^1^ Biomedical Engineering Laboratory, Escola Politécnica University of Sao Paulo Sao Paulo Brazil; ^2^ Department of Pharmacology, Institute of Biomedical Science University of Sao Paulo Sao Paulo Sao Paulo Brazil; ^3^ Unit of Pulmonary Pharmacology, School of Cancer and Pharmaceutical Sciences King's College London London UK

**Keywords:** forced oscillation, mice, respiratory mechanics, sedative, smooth muscle

## Abstract

Acepromazine (ACP) is one of the most used sedatives in veterinary medicine, and its cardiovascular effects are well documented. In contrast, its effects on the respiratory system remain less well understood, particularly in the context of assessment of respiratory mechanics in murine models of asthma, where its use has become more common in recent years. This study aims to investigate the effect of ACP on respiratory mechanics in BALB/c mice, specifically in an ovalbumin (OVA)‐induced model of allergic asthma. Mice received ACP (2.5 mg/kg) in combination with standard ketamine (100 mg/kg) and xylazine (10 mg/kg) anaesthesia. Respiratory mechanics were assessed in vivo using forced oscillation technique (FOT) following methacholine (MCh) challenge. Additionally, tracheal rings and lung strips were evaluated in vitro for airway smooth muscle (ASM) responsiveness to MCh. ACP administration significantly reduced Newtonian resistance (Rn) in OVA‐sensitised mice following MCh challenge, suggesting an inhibitory effect on ASM contractility. Furthermore, ACP mitigated increases in tissue damping (G) and hysteresivity (*η*), particularly in the inflamed lungs of OVA‐treated mice. In vitro, ACP induced a rightward shift in MCh concentration–response curves in non‐inflamed ASM and lung strips. These findings highlight the potential confounding effects of ACP in studies assessing airway hyperresponsiveness and offer insights into how sedative choice can shape the scientific validity of studies involving assessment of respiratory mechanics.

## 1 Introduction

The forced oscillation technique (FOT) is widely employed to understand the mechanisms of lung disease or the effects of drugs on the respiratory system in small rodents. FOT involves introducing a broadband flow perturbation into the respiratory system and recording the resulting pressure signal [1]. These signals are then used to estimate respiratory system impedance and fitting it to the constant phase model (CPM), originally introduced by Hantos and colleagues [2]. The experimental setup requires tracheotomy and controlled mechanical ventilation, often exceeding 30 min, which requires a reliable and robust anaesthesia protocol and inhibition of spontaneous breathing [3, 4], primarily because breathing effort significantly influences the assessment of respiratory mechanics [5]. Hence, adequate anaesthesia induction is not only essential for ensuring procedural success but also plays a critical role in preserving physiological stability, minimising animal distress, and maintaining data integrity. Importantly, the quality and type of sedation can significantly influence respiratory mechanics parameters, making the choice of drugs a crucial variable in experimental design [4]. There is not a consensus regarding the best choices for anaesthetic induction but a robust amount of literature evidence that a cocktail of ketamine/xylazine has been the first choice by research groups over the years [6, 7, 8]. To note that none of those studies have discussed the success of the protocol in bringing animals to an adequate level of anaesthesia or the percentage of mortality.

Optimising anaesthetic protocols aligns with the ethical framework of the 3Rs (Replacement, Reduction, and Refinement). In addition, Refinement focuses on minimising pain and distress while improving scientific outcomes. Enhancing anaesthesia protocols is thus important for Refinement, promoting animal welfare and increasing the reliability of physiological measurements [9, 10]. Hence, sedatives that provide adequate analgesia and immobility without compromising key respiratory parameters are important for generating ethically responsible and scientifically valid data.

Acepromazine (ACP), a phenothiazine derivative commonly used as a sedative in veterinary medicine [11], has been integrated into anaesthetic protocols for respiratory mechanics assessment in small animal studies [12, 13, 14, 15]. This practice is supported by reports highlighting its favourable safety profile and its ability to enhance and prolong the anaesthetic effects of ketamine/xylazine combinations [16, 17, 18, 19]. To our knowledge, there is no report of its impact on respiratory mechanics, particularly in the context of airways hyperresponsiveness in allergic asthma. Given ACP's pharmacodynamic actions on muscarinic, adrenergic, and histaminergic receptors, it may influence respiratory mechanics beyond sedation [19, 20], potentially confounding experimental outcomes.

This study explores the effects of ACP on respiratory mechanics in an experimental model of allergic asthma.

## 2 Results

### 2.1 Leukocytes in BAL

**FIGURE 1:**
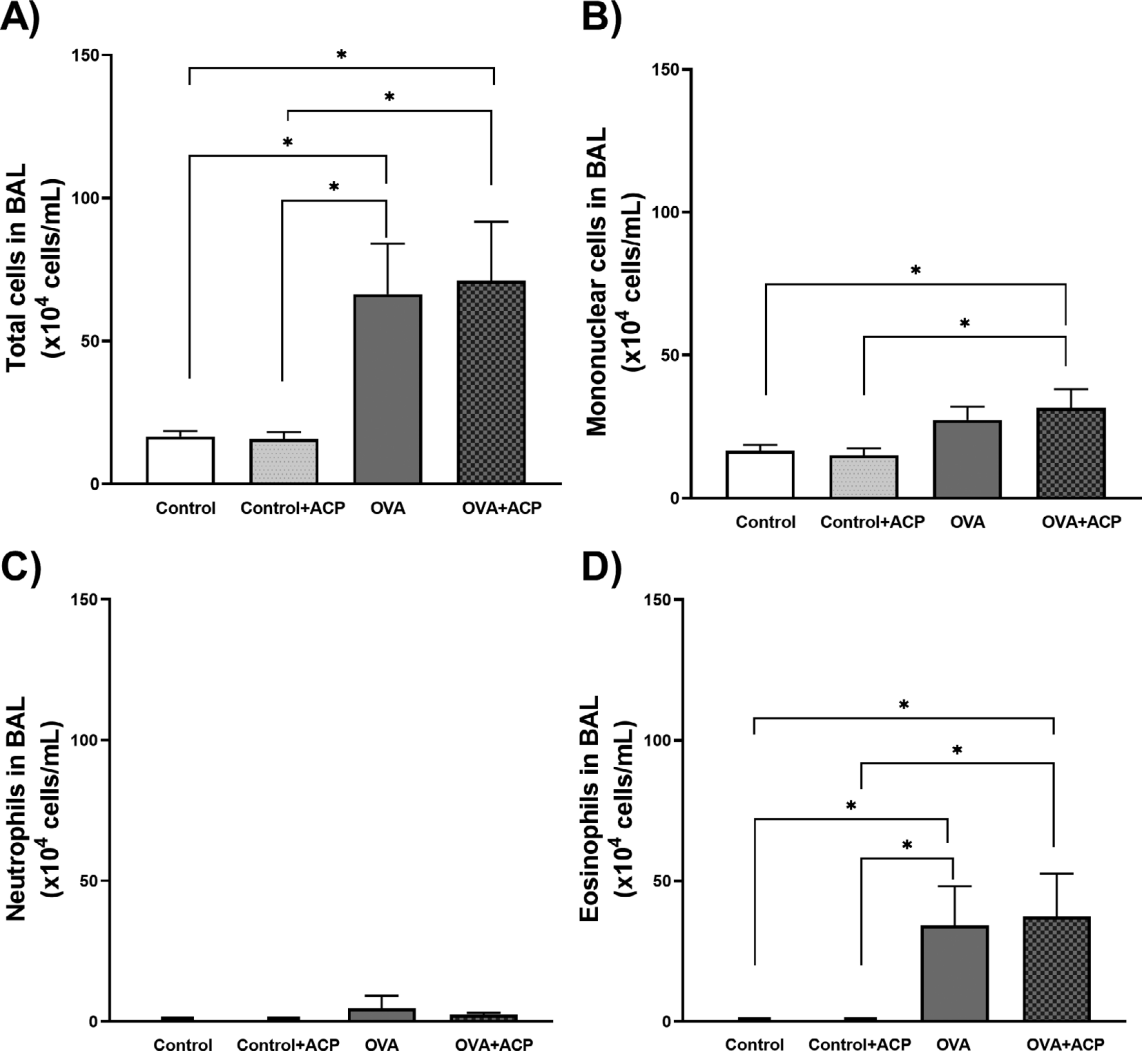
Effect of antigen‐induced lung inflammation on total and differential leucocyte count in bronchoalveolar lavage (BAL) of BALB/c mice across groups: Control (*n* = 12), control + ACP (*n* = 12), OVA (*n* = 12) and OVA + ACP (*n* = 11). Panel (A) shows the total leucocyte counts in BAL. Panels (B–D) present the differential counts for macrophages, neutrophils, and eosinophils, respectively. The results are expressed as Mean ± SD. Significant differences among groups were analysed by two‐way ANOVA, followed by Tukey post‐test. *p < 0.05.

Our OVA protocol induced a significant increase in eosinophil numbers in the OVA ($66.3\pm 15.6\;10^{4}$ cell/mL) and OVA + ACP ($71.1\pm 17.8\;10^{4}$ cell/mL) groups compared with the control ($16.5\pm 2.0\;10^{4}$ cell/mL) and control + ACP ($15.7\pm 2.6\;10^{4}$ cell/mL) groups, as illustrated in Figure 1. To note there was no difference in total cells nor eosinophils between the OVA and OVA + ACP group.

### 2.2 Effect of Acepromazine on Airway Resistance

**FIGURE 2:**
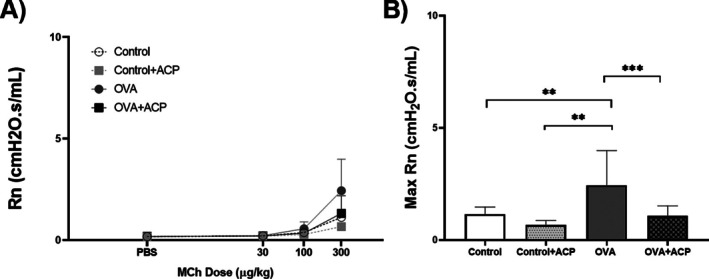
The dose–response curve of airway resistance (Rn) to increasing doses of methacholine (MCh) in BALB/c mice (*n* = 11 per group) (A). Max Rn values were used to compare between groups (B). Data are expressed as Mean ± SD. Significant differences among groups were analysed by two‐way ANOVA, followed by Tukey post‐test. **p < 0.01; ***p < 0.001.

In all groups, baseline values of airway resistance (Rn) presented no variations. As presented in Figure 2, OVA mice had significantly higher values of $R_{n}$ ($2.44\pm 1.45\;{\mathrm{cmH}}_{2}O\;s\;{\mathrm{mL}}^{-1}$, *p* < 0.01) compared to OVA+ ACP ($1.07\pm 0.44\;{\mathrm{cmH}}_{2}O\;s\;{\mathrm{mL}}^{-1}$). Notably, OVA+ACP group had a lower Rn response that was not different from the control group.

### 2.3 Effect of Acepromazine on Elastance

**FIGURE 3:**
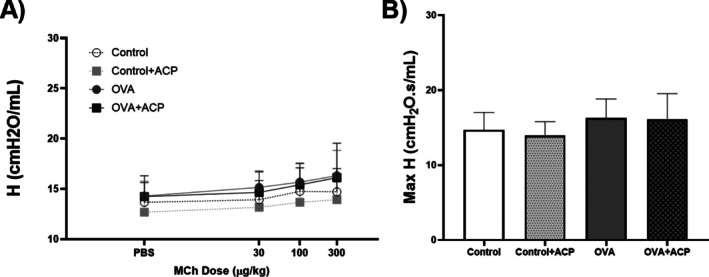
Elastance (H) changes in response to increasing doses of methacholine (MCh) across groups (*n* = 11 per group) (A). Panel (B) shows the max H values at the last dose of MCh. Data are expressed as Mean ± SD. Significant differences among groups were analysed by two‐way ANOVA, followed by Tukey post‐test.

There were no changes in baselines values of elastance (H) across the groups (Figure 3). Likewise, after MCh challenge there were not significant variations.

### 2.4 Effect of Acepromazine on Tissue Damping

**FIGURE 4:**
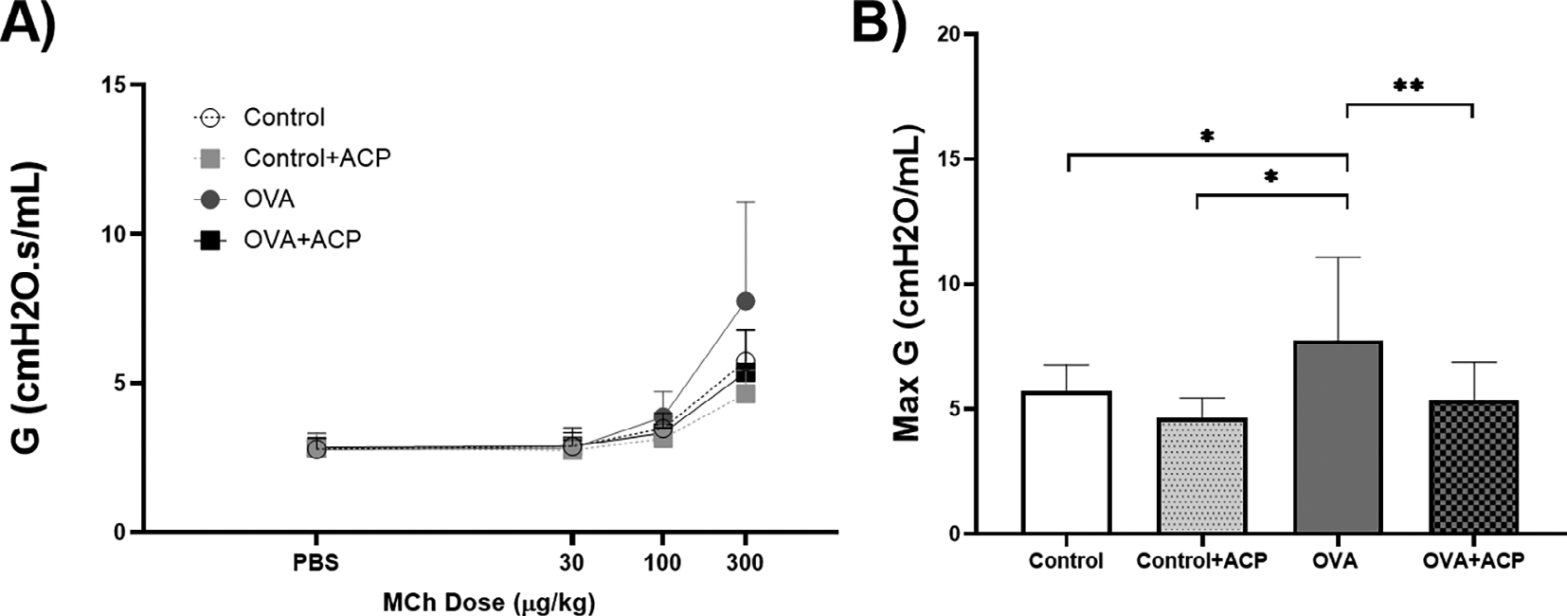
The dose–response curve of tissue damping (G) to increasing doses of methacholine (MCh) in BALB/c mice (*n* = 11 per group) (A). Max Rn values were used to compare between groups (B). Data are expressed as Mean ± SD. Significant differences among groups were analysed by two‐way ANOVA, followed by Tukey post‐test. *p < 0.05; **p < 0.01.

The last methacholine challenge exerted significant variations of tissue damping (G). OVA‐sensitised mice had higher values when compared to the other groups (Figure 4). No significant differences were observed among the other groups.

### 2.5 Effect of Acepromazine on Hysteresivity

**FIGURE 5:**
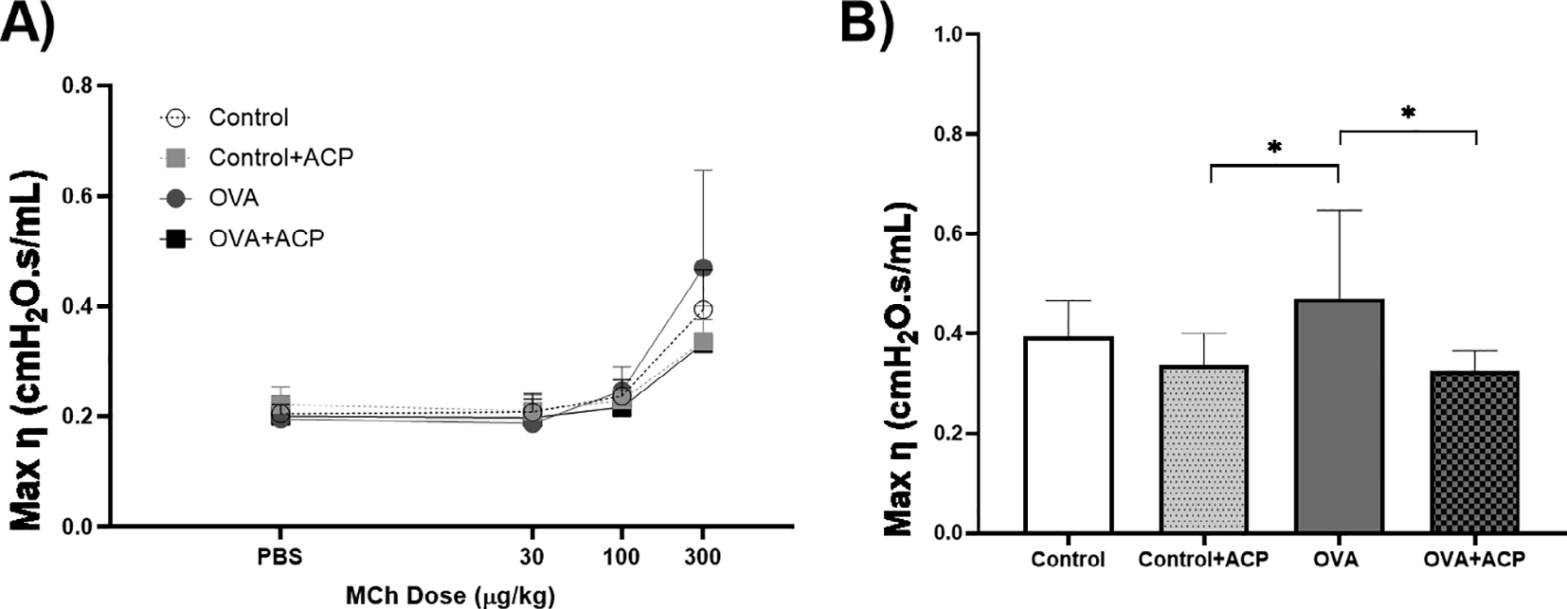
Effect of acepromazine (ACP) on hysteresivity (*ƞ*) in an OVA‐sensitised model of asthma in BALB/c mice (*n* = 11 per group) (A). Max *ƞ* was employed to compare changes among groups. Data are expressed as Mean ± SD. Significant differences among groups were analysed by two‐way ANOVA, followed by Tukey post‐test. *p < 0.05.

To further understand the effect of ACP in respiratory mechanics, we estimated hysteresivity (ƞ), which is related to heterogeneity in the respiratory system. Interestinlgy, groups receiving ACP: Control + ACP (0.33±0.10) and OVA + ACP (0.32±0.06) mice had the lower values of ƞ and there were significant differences when compared to OVA group (0.47±0.17). This is illustrated in Figure 5.

### 2.6 Histological Analysis

**FIGURE 6:**
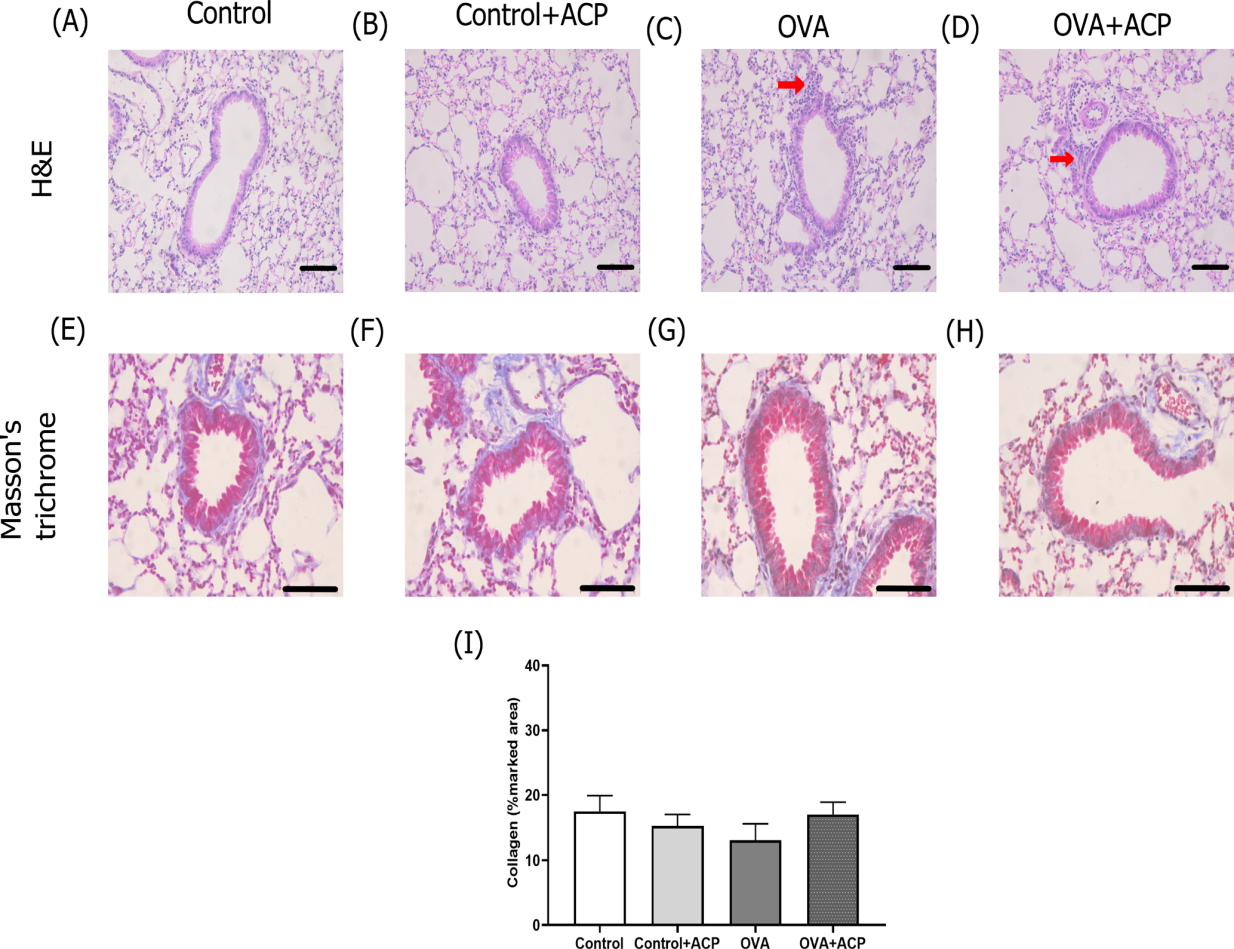
Effect of OVA protocol on lung inflammation in mice. Representative images of haematoxylin/eosin (H&E) staining in lung slices are depicted in panels (A–D) (scale bar 50 μm). Leukocyte infiltrates are observed in lung sections (C, D) (red arrows) in OVA groups. In panels (E–H), representative images of lungs stained with Masson's trichrome are depicted (scale bar 100 μm). In (I), collagen density was not change across groups (*n* = 5 per group).

The effect of OVA treatment impairing leukocyte infiltration into the lungs are depicted in Figure 6 in H&E images (Figure 6c,d). In addition, no change in collagen deposition was observed across groups by Masson's trichrome analysis (Figure 6E–H).

### 2.7 Effect of Acepromazine on Trachea and Lung Strip Reactivity

**FIGURE 7:**
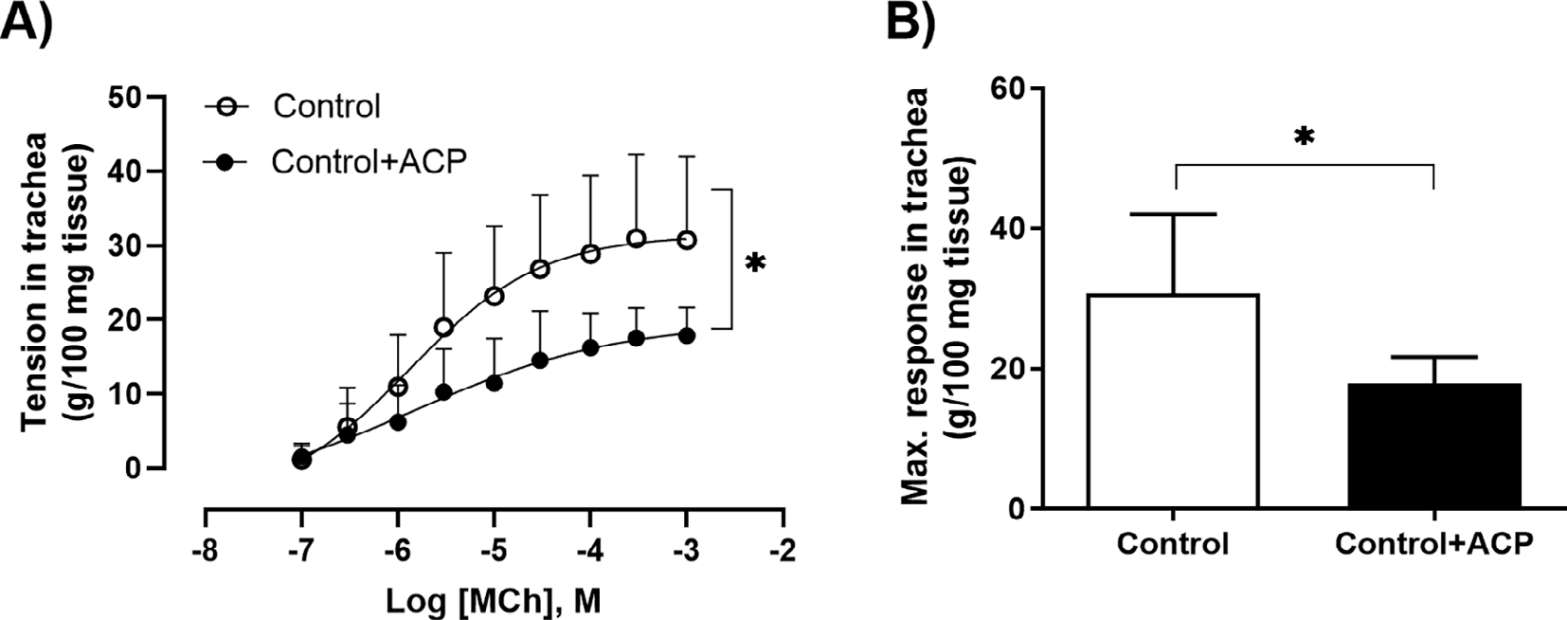
Effect of acepromazine on methacholine‐induced contraction in murine tracheal rings (*n* = 7 per group). Tracheal rings were exposed to cumulative concentrations of methacholine (MCh) ($1\times 10^{-7}\;M$ to $1\times 10^{-3}\;M$), with or without preincubation with acepromazine (ACP) ($3\times 10^{-7}\;M$, 1 h). ACP significantly reduced the contractile response to MCh, particularly at the highest concentration ($1\times 10^{-3}\;M$). Data are presented as mean ± SD. *p < 0.05.

MCh induced stable, concentration‐dependent contractions in murine tracheal rings of non‐allergic mice. Preincubation with ACP at a concentration of $3\times 10^{-7}\;M$ attenuated the contractile response to MCh, as illustrated in Figure 7.

**FIGURE 8:**
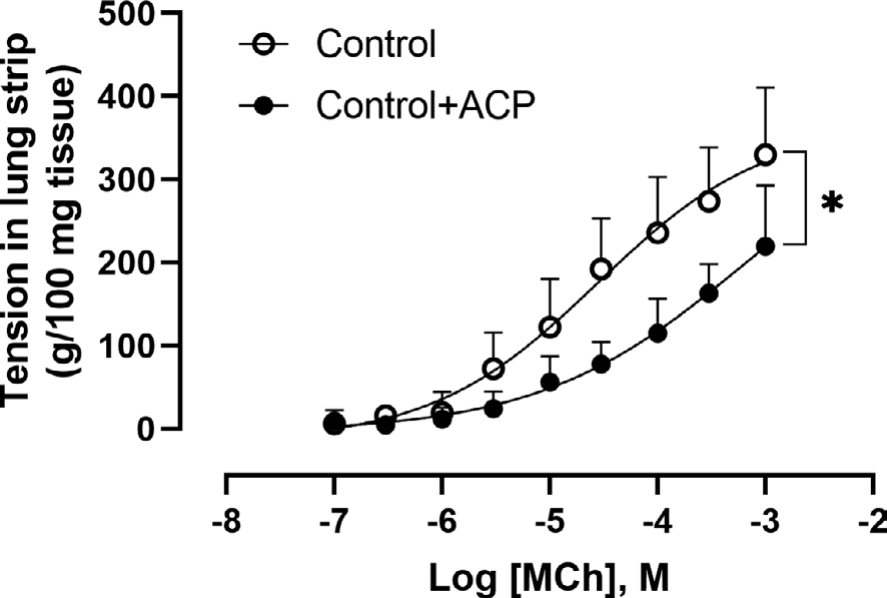
Effect of acepromazine on methacholine‐induced contraction in murine lung strips (*n* = 7 per group). Lung strips were exposed to cumulative concentrations of methacholine (MCh) ($1\times 10^{-7}\;M$ to $1\times 10^{-3}\;M$), with or without preincubation with acepromazine (ACP) ($3\times 10^{-7}\;M$, 1 h). ACP significantly reduced the contractile response to MCh, particularly at the highest concentration ($1\times 10^{-3}\;M$). Data are presented as mean ± SD.*p < 0.05.

Likewise, MCh exerted consistent concentration‐dependent contractions in lung strips. Pretreatment with ACP at a concentration of $3\times 10^{-7}\;M$ significantly reduced the tissue's responsiveness to MCh, as shown in Figure 8.

## 3 Discussion

ACP is part of the phenothiazine derivatives which present some degree of affinity for muscarinic, adrenergic and histaminic receptors leading to peripheral effects [21, 22, 23]. Especially, ACP impairs cardiovascular function; for instance, it impairs peripheral vasodilation due to *α*1 adrenoceptors blockage [19, 20]. Regarding ACP effects on respiratory mechanics, little has been described over the years. Notably, ACP has been reported to cause a decrease in respiratory rate in rabbits [24], dogs [25] and sheep [26]. However, to our knowledge, there are no studies on its respiratory peripheral effects. We pursued this study to determine whether the use of ACP alters respiratory mechanics in a model of allergic asthma.

In our model of allergic asthma, OVA animals had a higher lung inflammation (Figure 1) and presented hyperresponsiveness to MCh at a dose of 300 μg/kg (Figure 2). This pattern is consistent with previous reports [8, 27, 28] indicating that more robust and statistically meaningful changes in respiratory mechanics occur at higher MCh doses. The absence of differences at lower methacholine doses is expected as these concentrations are insufficient to produce a strong and sustained bronchoconstrictor response in vivo. Also, due to the acuteness of the protocol we did not observe changes in collagen deposition (Figure 6). Our results evidenced that ACP reduced Rn in the respiratory mechanics assessment of OVA‐induced allergic asthma. The reduction of Rn may be explained by an ACP direct effect on ASM impairing contraction response to MCh. We hypothesized that this effect could be mediated by ACP's anticholinergic properties as previous reports on other phenothiazine derivatives (LG 30435) described an MCh‐ and histamine‐inhibition of trachea contraction [29] but no similar report regarding ACP effect was found. In addition, other phenothiazine derivatives (promethazine and mequitazine) showed affinity for muscarinic receptors [30, 31].

In Figures 2 and 4, the effects of ACP in Rn and G in the control + ACP group showed a non‐significant but sustained reduction compared with the control group, suggesting an effect on tissue contraction in non‐allergic animals. To further investigate this, we examined the effect of ACP on tracheal rings and lung strips from non‐allergic mice only. OVA‐sensitised mice were not included, as airway smooth muscle (ASM) hypercontractility in asthma models is thought to be primarily driven by the inflammatory and structural environment of the asthmatic lung rather than by intrinsic abnormalities in ASM. Supporting this, in vitro experiments using tracheal rings from BALB/c and C57BL/6 mice demonstrated that pre‐incubation with LPS or inflammatory cytokines such as TNF*α* enhances contraction [32]. Similarly, increased ASM contraction in response to TNF*α* has been reported in guinea pigs and pigs [33, 34]. More recently, this cytokine‐induced hypercontractility has been linked to activation of the PKC–Src pathway [35]. In both of our preparations, ACP induced a rightward shift in the concentration–response curve to MCh, suggesting that ACP inhibits MCh‐induced ASM contraction in both the trachea and lung parenchyma. This effect may explain the reduced values of Rn and G not only in OVA‐sensitised mice but also in Control mice, reflecting the ACP's action on ASM in both central and peripheral airways.

In murine experiments assessing respiratory mechanics using MCh, the parameter *ƞ* increases as heterogeneity in the lungs increases [36]. Accordingly, increasing concentrations of MCh, combined with airway shunting, led to elevated ƞ values [37], a trend most pronounced in the OVA group (Figure 5). Interestingly, the *ƞ* values for the control + ACP and OVA + ACP groups were relatively similar, 0.32 and 0.33, respectively, suggesting that ACP may reduce the development of airway heterogeneity. Regarding other parameters of the CPM, the *G* value following the final dose of MCh in the OVA group was significantly higher compared to the other groups. This increase likely reflects either changes in tissue resistance or heightened heterogeneity in the peripheral airways due to contraction, as *G* is closely associated with tissue heterogeneity [38, 39]. Notably, in the OVA group, *G* increased by approximately 200% between the 100 and 300 mg/kg MCh doses, while the increase was around 157% in the other groups. Conversely, no significant changes in the *H* parameter were observed across groups, consistent with our protocol, which did not induce alterations in lung parenchyma or chest wall stiffness [40].

Several limitations of this study should be considered. First, while our results indicate a possible role for muscarinic receptors, we did not determine the specific receptor subtypes involved or the associated intracellular signalling pathways. Further investigations assessing the effect of specific receptor activation, ACP‐induced changes in neurotransmitter release, PKC–Src signalling or calcium release could help elucidate the mechanisms underlying these effects. Second, in vivo studies commonly administer ACP at doses ranging from 2 to 10 mg/kg. We selected a 2.5 mg/kg dose because, when used in combination with ketamine and xylazine, it provides reliable anaesthetic induction while minimising potential cardiovascular effects [16, 18, 19, 20]. Nonetheless, we acknowledge that the dose–response relationship of ACP was not examined in this study, and future research should address the effect of higher doses on respiratory mechanics. Lastly, the possible interaction of the ketamine/xylazine cocktail with ACP deserves further research as it is known that it may cause hypotension, hypoxia and bradycardia [19]. Although the doses of ketamine and xylazine used in our protocol fall within standard ranges designed to minimise these adverse effects [4], we cannot entirely exclude the possibility of pharmacodynamic interactions. Such interactions may have influenced the respiratory mechanics parameters observed and should therefore be considered when interpreting our findings.

Our findings have important methodological implications, particularly given the increasing use of ACP in anaesthesia protocols for respiratory mechanics studies. We demonstrate that ACP attenuates airway hyperresponsiveness, which may confound assessments of respiratory mechanics in mice and potentially compromise the accuracy and interpretation of experimental outcomes. The study design enabled evaluation of ACP effects on airway hyperresponsiveness both in vivo and in vitro, providing complementary insights from central airways to peripheral lung structures. Moreover, the use of the OVA model of allergic asthma allowed us to examine the effects of ACP on airway hyperresponsiveness in both inflamed and non‐inflamed lung tissue.

## 4 Materials and Methods

### 4.1 Animals

Animal protocols were approved by the Ethical Committee of the Institute of Biomedical Science of the University of Sao Paulo (CEUA 11952008). The study was conducted in male BALB/c mice. The animals were maintained in the biological service unit of the Pharmacology Department in a temperature‐controlled room under a 12 h/12 h light/dark cycle. Mice had free access to rodent chow and tap water.

### 4.2 Antigen‐Induced Allergic Inflammation

14‐week‐old BALB/c mice were immunised with two subcutaneous injections (SI) of chicken egg ovalbumin (OVA) (Sigma, USA) emulsified with aluminium hydroxide on days 1 and 7, the OVA concentration for the SI was 30 and 50 μg in a volume of 0.2 mL, respectively. Afterwards, mice received three intranasal challenges with 30 μL of solution containing 10 μg of OVA on day 14 and 20 μg of OVA on Days 21 and 28. Sham mice underwent the same protocol but were treated with sterile PBS.

### 4.3 Experimental Design

#### 4.3.1 Sedation and Anaesthesia

Control‐ and OVA‐sensitised mice were randomly divided into four groups: control, control + ACP, OVA, OVA + ACP. The ACP groups received an intraperitoneal (IP) injection of sterile PBS or ACP (2.5 mg/kg, Syntec, Brazil) 30 min before the experiment. Later, mice receiving ACP were anaesthetised with an IP injection of a cocktail of ketamine (100 mg/kg, Syntec, Brazil) and xylazine (10 mg/kg, Ceva, Brazil). The other groups received a cocktail of ketamine (120 mg/kg, Syntec, Brazil) and xylazine (12 mg/kg, Ceva, Brazil). The tail, pedal, and paw reflexes were assessed to guarantee adequate anaesthesia induction.

#### 4.3.2 Assessment of Respiratory Mechanics

Mice were located in a supine position below a light heater to guarantee body temperature maintenance. Tracheotomy was performed with an 18G metallic cannula (BD Company, Brazil). Then, mice were connected to a small animal ventilator (SAV) (flexiVent, SCIREQ, Canada). Continuous mechanical ventilation was provided with the following setup: Positive end‐expiratory pressure (PEEP) of 3 cmH_2_O, tidal volume of 10 mL/kg, and a respiratory rate of 250 breaths/min. An IP injection of pancuronium bromide (1 mg/kg, Cristália, Brazil), a neuromuscular blockade drug, was given to optimise ventilation, diminish discomfort, and perform the assessment of the respiratory system. Once the ventilation pattern stabilised, the jugular vein was cannulated to inject acetyl‐*β*‐methyl‐choline chloride (MCh) (Sigma‐Aldrich, USA), an agonist commonly used to evaluate respiratory mechanics in murine models of allergic asthma [36]. The initial measurement of these parameters occurred following the injection of 20 μL/10 g body weight of phosphate‐buffered saline (PBS), which serves as a diluent for MCh, administered through the jugular vein. Subsequent measurements were conducted following additional doses of MCh (30, 100, and 300 μg/kg) in a volume equivalent to 20 μL/10 g body weight.

### 4.4 Lung Histology

Whole lungs were inflated with 4% paraformaldehyde. The lobes were separated and embedded in paraffin wax with random orientations. Embedded lungs were cut into 5‐μm‐thick sections employing a microtome. Tissue sections were mounted on slides and stained with Haematoxylin and Eosin stain (H&E) (Sigma‐Aldrich, USA) and Masson's trichrome (Abcam, UK). The stained slides were randomly sampled and photographed using a light microscope (Leica DM 2000, Germany) at ×20 magnification. The collagen density was assessed using image processing software [41].

### 4.5 Total and Differential Cell Counts

Lungs were flushed out two times with 0.8mL of PBS through a tracheal cannula. BAL was pooled and centrifuge (1500 rpm, 15 min, at 4°C). The pellet was resuspended in 1 mL of PBS and total cell in the BAL were counted using a hematocytometer. To perform differential cell counts, the BAL suspension was centrifuged in a cytospin and was subsequently stained with Diff Quick (NewProv, Brazil). A total of 200 cells were then counted using light microscopy (Leica DM 2000, Germany).

### 4.6 Lung Strips and Trachea Reactivity

The upper tracheal rings were isolated and stored in cold Krebs solution (NaCl 115, KCl 4.6, CaCl_2_·2H_2_O 2.5, KH_2_PO4 1.2, MgSO_4_·7H_2_O 2.5, NaHCO_3_ 25, and glucose 11) for later analysis. Lungs were perfused with Krebs solution to remove residual blood. The left lung was then isolated, and a strip measuring approximately 2 mm × 10 mm was excised using callipers for precise measurement. Each end of the lung strip was dried and adhered to a support using tissue adhesive, with a setting time of 20 s per end. Lung strips were mounted in an organ bath containing 15 mL of Krebs solution, maintained at 37°C and continuously gassed with a gas mixture of 95% O_2_ and 5% CO_2_. The strips were suspended using metal wires, with one end attached to a 3d printed fixed support and the other to a force transducer. A resting tension of 1 g was applied, and tissues were allowed to equilibrate for 45 min, with Krebs solution refreshed every 15 min. Upper tracheal rings were carefully dissected and mounted in a similar organ bath setup as described for lung strips. The same aeration and temperature conditions were maintained. After mounting, rings were stabilised under an appropriate resting tension for 45 min with Krebs solution replaced every 15 min. Data was recorded using a computer‐based acquisition system (PL3508 PowerLab, ADinstruments, New Zealand) and a force transducer (MLT0420, ADInstruments, New Zealand), then data was processed with a dedicated software (LabChart, ADInstruments, New Zealand).

Concentration‐response curves to methacholine (100 nM–1 mM) were performed in the presence and absence of ACP (300 nM, 1 h prior to methacholine stimulation) for 1 h prior to MCh stimulation.

Following the methacholine challenge, tissues were washed three times at 10‐min intervals with fresh Krebs solution. Depolarization with potassium chloride (KCl, 60 mM) was performed. Lung strips were then fixed in 4% formaldehyde for histological processing to check tissue viability by excluding strips containing larger airways.

### 4.7 Statistical Analysis

Statistical analysis was conducted with a statistical package (Prism 10, GraphPad Software Inc., USA). Data is reported as mean ± SD. Normality tests were performed in all the data. One‐way ANOVA with Bonferroni's post hoc test was done to determine statistical significance. In vitro data analysis was done using Student's *t*‐test.

## Author Contributions

All authors contributed to data collection and review of the document. Furthermore, Alembert Lino‐Alvarado and Henrique Takachi Moriya took charge of conducting the literature search, formulating the study design, and preparing the manuscript.

## Funding

This study was financed in part by the Conselho Nacional de Desenvolvimento Científico e Tecnológico (CNPq—Brazilian National Council for Scientific and Technological Development)—Brazil (309464/2022‐6) and the Coordenação de Aperfeiçoamento de Pessoal de Nível Superior—Brazil ‐ Finance Code 001 (CAPES) (grant number 88887.620568/2021‐00). Rafael Campos thanks Fundação de Amparo à Pesquisa do Estado de São Paulo (grant number FAPESP 2023/15350‐9). The Article Processing Charge for the publication of this research was funded by the Coordenação de Aperfeiçoamento de Pessoal de Nível Superior ‐ Brasil (CAPES) (ROR identifier: 00x0ma614).

## Conflicts of Interest

The authors declare no conflicts of interest.

## Data Availability

The data that support the findings of this study are available from the corresponding author upon reasonable request.
